# A266 DIET-BASED THERAPIES FOR INTESTINAL DYSFUNCTION INDUCED BY CLOSTRIDIOIDES DIFFICILE INFECTION

**DOI:** 10.1093/jcag/gwac036.266

**Published:** 2023-03-07

**Authors:** Z Saqib, X Bai, Y Nishihara, J Lu, G De Palma, P Bercik, S Collins

**Affiliations:** 1 Medicine; 2 McMaster University, Hamilton, Canada

## Abstract

**Background:**

Chronic gut dysfunction occurs in up to 25% of patients following antibiotic-treated C. *difficile* infection (CDI). We developed a humanized mouse model in which germ free mice colonized with microbiota from patients with severe constipation post-CDI developed slow colonic transit, as a result of damage to the Interstitial Cells of Cajal (ICC) network by pro-inflammatory macrophages. Colonic transit, immune activation and the ICC network normalized after fecal microbiota transplantation using samples from healthy mice, as well after treatment with psyllium fiber. Here we explored the long-term effects of psyllium and evaluated the therapeutic potential of pectin and quercetin in this model.

**Purpose:**

**1**) To investigate the time course of the beneficial effect of psyllium on colonic motility.

**2**) To explore possible therapeutic properties of flavonoids and pectin.

**Method:**

Germ-free mice were colonized with microbiota from the post-CDI (PCDI) patient or healthy controls (HC). After 3 weeks, the mice were fed for 4-5 weeks with a control diet or diets with 15% psyllium (PSY), 10% pectin (PCT) or 0.05% quercetin (QCT). To evaluate time course of PSY on motility, control diet was administered for 3-weeks following PSY treatment. The bead expulsion test was used to assess colonic motility. Stool samples were collected for microbial profiling, and short and branched-chain fatty acids (SCFA/BCFA) analysis. Macrophages morphology and counts, and ICC network structure were evaluated by immunohistochemistry.

**Result(s):**

Compared to HC microbiota, colonization with post-CDI microbiota induced slow colonic transit in recipient mice, and this was normalized by PSY (n=13; p=0.02). The benefit of PSY was transient as colonic transit slowed following discontinuation of PSY (p=0.001). The changes in colonic transit were paralleled by switch in macrophages phenotype and damage to the ICC network. Additionally, discontinuation of PSY resulted in a return of microbial diversity (p< 0.001), SCFA/BCFA levels (acetic and propionic acid/ iso-butyric and valeric acid) and specific bacterial species abundances, to values seen in untreated mice colonized with post-CDI microbiota. Microbial analysis predicted potential pathways involved in macrophage polarization, including the synthesis of SCFA/BCFA, degradation of inositol and production of acetylglucosamine.

PCT also normalized slow intestinal transit in mice colonized with post-CDI microbiota (p=0.003), restored phenotype of infiltrating macrophages, and improved the structural integrity of the ICC network. In contrast, QCT failed to improve gut dysfunction in PCDI mice.

**Conclusion(s):**

Our results suggest that the beneficial effects of psyllium in this model are transient. Dietary pectin, but not quercetin, may also serve as a novel treatment strategy to restore colonic motility and immune homeostasis in humans with severe constipation post-CDI.

**Please acknowledge all funding agencies by checking the applicable boxes below:**

Other

**Please indicate your source of funding;:**

W. Garfield Weston Foundation

**Disclosure of Interest:**

None Declared

